# Intramuscular uptake of tranexamic acid during haemorrhagic shock in a swine model

**DOI:** 10.1186/s13049-021-00983-2

**Published:** 2021-12-18

**Authors:** Håkon Kvåle Bakke, Ole Martin Fuskevåg, Erik Waage Nielsen, Erik Sveberg Dietrichs

**Affiliations:** 1 Department of Anaesthesia and Critical Care, University Hospital of North Norway, 9038 Tromsø, Norway; 2 Department of Traumatology, University Hospital of North Norway, Tromsø, Norway; 3 Department of Health and Care Sciences, Faculty of Health Science, UiT, The Arctic University of Norway, Tromsø, Norway; 4 Division of Diagnostic Services, University Hospital of North Norway, Tromsø, Norway; 5 Department of Anaesthesia and Critical Care, Nordland Hospital, Bodø, Bodø, Norway; 6 University Nord, Bodø, Norway; 7 Department of Immunology, University of Oslo, Oslo, Norway; 8 Institute of Clinical Medicine, UiT, The Arctic University of Norway, Tromsø, Norway; 9 Experimental and Clinical Pharmacology, Department of Medical Biology, UiT, The Arctic University of Norway, Tromsø, Norway; 10 Center for Psychopharmacology, Diakonhjemmet Hospital, Oslo, Norway

**Keywords:** Injury, Trauma, Bleeding, Coagulation

## Abstract

**Background:**

Tranexamic acid (TXA) reduce mortality in bleeding trauma patients, with greater effect if administered early. Serum concentrations above 10 µg/mL are considered sufficient to inhibit fibrinolysis. Normally administered intravenously (i.v.), TXA can also be administered intramuscularly (i.m.). This could be advantageous in low resource and military settings, if sufficient serum concentrations can be reached in shocked patients with reduced muscular blood perfusion. Accordingly, we aimed to: (1) Determine the impact of shock on the pharmacokinetics of i.m. TXA, and (2) Compare the pharmacokinetics of i.v. versus i.m. TXA in ongoing shock.

**Materials and methods:**

In a prospective experimental study, N = 18 Norwegian landrace pigs (40–50 kg), utilised in a surgical course in haemostatic emergency surgery, were subjected to various abdominal and thoracic trauma. After 1 h of surgery the animals were given 15 mg/kg TXA either i.v. or i.m. A control group without injury, or surgery, received intramuscular TXA. Blood samples were drawn at 0, 5, 15, 25, 35, 45, 60 and 85 min. The samples were centrifuged and analysed with liquid chromatography–tandem mass spectrometry (LC–MS/MS) for TXA serum-concentrations.

**Results:**

In shocked pigs, i.m. administration resulted in a mean maximum serum concentration (C_max_) of 20.9 µg/mL, and i.v. administration a C_max_ of 48.1 µg/mL. C_max_ occurred 15 min after i.m. administration and 5 min after i.v. administration. In non-shocked swine, i.m. administration resulted in a C_max_ of 36.9 µg/mL after 15 min. In all groups, mean TXA serum concentrations stayed above 10 µg/mL from administration to end of experiments.

**Conclusions:**

I.m. administration of TXA in shocked pigs provides serum concentrations associated with inhibition of fibrinolysis. It may be an alternative to i.v. and intraosseous administration during stabilisation and transport of trauma patients to advanced medical care.

## Background

Tranexamic acid (TXA) has been shown to reduce mortality in bleeding trauma patients [1]. As a synthetic lysine analogue, it works by inhibiting the activation of plasminogen to plasmin, and thereby fibrinolysis, thus enhancing clot stability and improving haemostasis [2]. In vitro studies have suggested that effective serum concentrations are in the range of 10–17.5 µg/mL [3–5]. The effect on mortality is time-dependent, with a 10% reduction in effect for every 15 min delay, and possibly with adverse effect if administered after more than 3 h [6, 7].

While TXA normally is administered intravenously (i.v.), it has been established that the intramuscular (i.m.) route is a feasible alternative. Absorption after iv. administration is faster, with higher peak serum concentrations (C_max_) than i.m. administration [8]. However, in certain environments i.m. administration would be advantageous. For example, combat medicine, or in low resource settings in which ambulance crews are not trained in i.v. catheter placement [9].

Prior to the beginning of this study, studies on i.m. uptake of TXA had only been conducted in healthy volunteers [8, 10, 11], whereas the patients eligible for TXA are in haemorrhagic shock or pre-shock [1]. Haemorrhagic shock leads to disturbances of skeletal muscle microcirculation, and muscular uptake of TXA is likely to be inhibited [12, 13] Therefore, we wanted to explore whether i.m. administered TXA provides sufficient uptake during haemorrhagic shock to achieve fibrinolysis-inhibiting serum concentrations.

Accordingly, the aims of this study were (1) To determine the impact of shock on the pharmacokinetics of i.m. TXA, and (2) to compare the pharmacokinetics of i.v. versus i.m. TXA in ongoing shock.

## Methods

### Model

Norwegian landrace pigs (n = 18), weighing 42–50 kg were used in the present study. Both male and female pigs were used, and assigned at random. All male pigs were castrated. The model consisted of animals primarily utilised in emergency trauma surgery courses, where surgical teams train in stabilisation of trauma patients. The courses, arranged by the Northern Norway Regional Health Authority has the following arrangement: The instructor inflicts intraabdominal injuries, and as the course progresses adds more and more injuries to the intra- and retro-abdominal organs, before finally inflicting intrathoracic injuries. The anaesthesia team attempts to stabilise the swine through administration of clear fluids, and noradrenaline.

### Animal preparation

The pigs were anesthetized directly at the farm, 10 min by car transport from the University lab. Anaesthesia was induced by azaperone 40 mg, ketamine 1000 mg and atropine 1 mg intramuscularly. After a peripheral ear vein catheter was established and 100 mg ketamine and 200 mg pentothal given i.v., pigs underwent tracheal intubation with an O.D. 7 mm tube and mechanically ventilated using a volume-controlled-ventilation (VCV), with a tidal volume of 10–15 mL/kg, rate of 20 per min. and a PEEP of 0 cm H_2_O. Inspiratory pressure was automatically adjusted by the ventilator to maintain tidal volume limits and to maintain an arterial pH 7.34–7.40. After intubation, anaesthesia was continued with intravenous infusion of morphine 2 mg/kg/h, midazolam 0.15 mg/kg/h and pentobarbital 4 mg/kg/h a. An arterial line for invasive pressure monitoring was placed in the right carotid artery. A pulse oximeter was placed on the ear, for continuous monitoring of peripheral oxygen concentration, and ECG-electrodes for continuous heart rate monitoring. The depth of anaesthesia was regularly controlled by Federation of European Laboratory Animal Science (FELASA)-certified qualified anaesthesia personnel. Pigs that were alive at the end of the experiment and emergency trauma course, were euthanised by pentobarbital 300 mg, morphine 10 mg, and potassium chloride 50 mmol.

### Experimental protocol

The animals were divided into three groups. A control group, *Group 1* (i.m., non-shock), without injury or surgery, received 15 mg/kg TXA (100 mg/mL) intramuscularly in the proximal, right thigh, immediately after establishment of the invasive lines. This group received no surgery. *Group 2* (i.v., shock) received 15 mg/kg TXA intravenously as a bolus in the peripheral ear vein catheter, 1 h after the start of surgery. *Group 3* (i.m., shock) received 15 mg/kg TXA intramuscularly in the proximal, right thigh, 1 h after the start of surgery. Start of surgery was defined as the time of first incision by the trauma course instructor. The animals were allocated to i.m. or i.v. at the beginning of the course, prior to surgery, and with the allocator blind as to which surgical team was assigned to which animal. For the consecutive day of the course, the teams that had operated on animals in group 2 were set to operate on an animal from group 3 and vice versa. The surgical teams were blinded to the administration form of TXA.

**Fig. 1:**
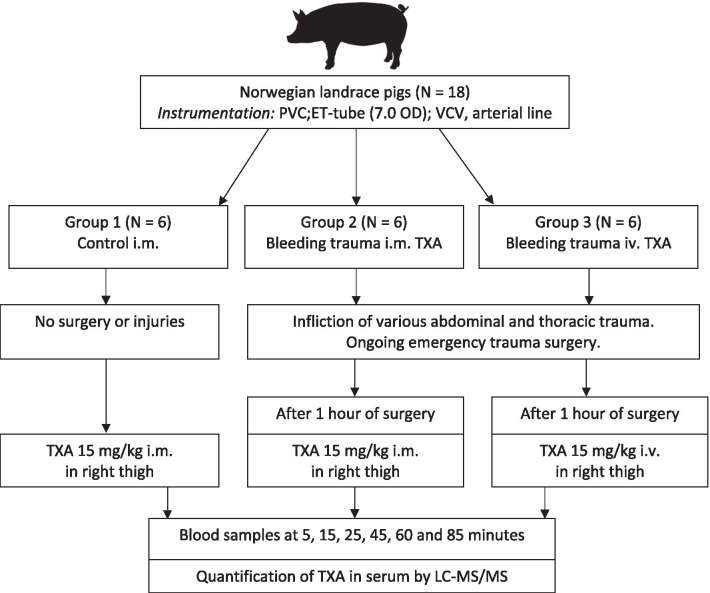
A general overview of the experimental protocol. *PVC* peripheral venous catheter, *ET* endotracheal, *OD* outer diameter, *VCV* volume-controlled-ventilation, *TXA* tranexamic acid, *LC–MS/MS* liquid chromatography–tandem mass spectrometry

Blood samples (2 mL full blood) were drawn prior to administration of TXA and at 5, 15, 25, 25, 45, 60 and 85 min. The samples were stored on Eppendorf tubes and set to coagulate for 1 h, before being cooled and centrifuged at 2500 × *g* for 7.5 min. The obtained plasma was frozen at – 20 °C prior to LC–MS/MS analysis. A general overview of the experimental protocol is provided as a flowchart in Fig. 1.

### Quantification of TXA concentrations

Quantification of TXA in serum was performed with liquid chromatography tandem mass spectrometry (LC–MS/MS). Stock solutions of 726 µg/mL TXA (Toronto Research Chemicals Inc. Ontario, Canada) was prepared in methanol:H_2_O (1:1) (Honeywell™ Riedel-de Häen™, Seelze, Germany) and stored at − 40 °C. A 7-point calibration curve was prepared by dilution of the stock solution with serum at the following concentrations: 94, 47, 9.4, 4.7, 0.94, 0.094 and 0.016 µg/mL. An internal standard solution was prepared by adding TXA-^13^C2,^15^N (Toronto Research Chemicals Inc. Ontario, Canada) to Milli-Q water (Millipore SAS, Molsheim, France) to a final concentration of 0.79 µg/mL.

Serum samples and spiked standards were prepared as follows: 50 µL sample, 50 µL internal standard solution and 700 µL methanol were mixed (5 s.) and centrifuged at 21,380 × *g* for 4 min. (Hettich Micro 200 centrifuge, Germany). 100 µL of supernatant was transferred to LC-vials. Samples were analysed by LC–MS/MS using a Waters Acquity UPLC *I*-Class FTN system with an autosampler and a binary solvent delivery system (Waters, Milford, MA) interfaced to Waters Xevo TQ-XS benchtop tandem quadrupole mass spectrometer (Waters, Manchester, UK). The mass spectrometer was operated in positive electrospray ion mode (ES+) and spray voltage was set to 0.85 kV. The system was controlled by MassLynx version 4.2 software. Desolvation gas temperature was 550 °C; source temperature was 150 °C; desolvation gas flow was 1000 L/h; cone gas flow was 150 L/h; collision gas pressure was 4 × 10^−3^ mBar (argon) and the ion energies were 0.5 V for both quadrupoles. The chromatography was performed on a 2.1 × 100 mm Waters Acquity Cortecs® T3, 1.6 µm column maintained at 50 °C. The injection volume was set to 0.1 µL. Eluent A consisted of 0.1% formic acid (Sigma-Aldrich, St. Louis, MO) in water; eluent B consisted of 0.1% formic acid in methanol. Gradient elution was performed with 1% B hold until 0.5 min and a linear increase to 70% B until 2 min, a linear increase to 98% B until 2.5 min, and re-equilibration until 3.6 min with 1% B with a flow rate at 0.30 mL/min. Column temperature was maintained at 50 °C and autosampler temperature was set to 4° C. For quantitative analysis of TXA, the following multiple reaction monitoring (MRM) transitions were used (bold transitions are qualifiers): m/z 158 → 123/**95** and 161 → 125/**96** (TXA and TXA-^13^C2,^15^N),

The method was validated and found to be linear from 0.005 to at least 94 µg/mL (r^2^ > 0.998) Lower limit of quantification (LLOQ) was found to be 0.0025 µg/mL (0.1 µL injection volume). Between-day coefficient of variation (CV) for TXA was < 10% on four consecutive days. CV for intraday precision value was < 6% and was calculated by assaying three samples (low, medium and high concentration) six times on the same day. Accuracy for recovery test was 94.2–106.2% (6 levels, n = 3 for each).

### Statistical analysis

Results are presented as mean ± standard deviation. Assessment for whether data were distributed normally was performed using Shapiro–Wilk test. Changes from start of the experimental protocol in hemodynamic variables and from peak TXA serum concentrations (C_max_), were compared by One-way repeated measures ANOVA. Problems with registering hemodynamic data from a few pigs distributed among the three groups, caused that hemodynamic average values are based on available data, on which statistical analysis was performed. When data were not normally distributed, repeated measures ANOVA on ranks was used. When significant differences were found, Dunnett’s method was used to compare values within group vs. baseline. Differences in TXA serum concentrations and hemodynamic variables between groups were analysed by a One-Way Analysis of Variance test followed by an all-pairwise multiple comparisons procedure using Tukey’s test. Repeated measures ANOVA on ranks and Dunn's test was used, when data were not normally distributed. Differences were considered significant at *p* < 0.05. Data are presented as mean ± standard deviation.

### Ethics

The research animals were registered in the Norwegian Food Safety Authority’s audit and applications system (Forsøksdyrsforvaltningens tilsyns- og søknadsystem, FOTS), and their use approved by the Norwegian Food Safety Authority (FOTS application number 15492) and the named animal care and welfare officer (Person med særskilt kontrollansvar, PMSK).

## Results

A total of 18 pigs were studied. The non-shock i.m. (Group 1), the i.v. shock group (Group 2), and the i.m. shock group (Group 3), all included 6 animals each.

**Fig. 2:**
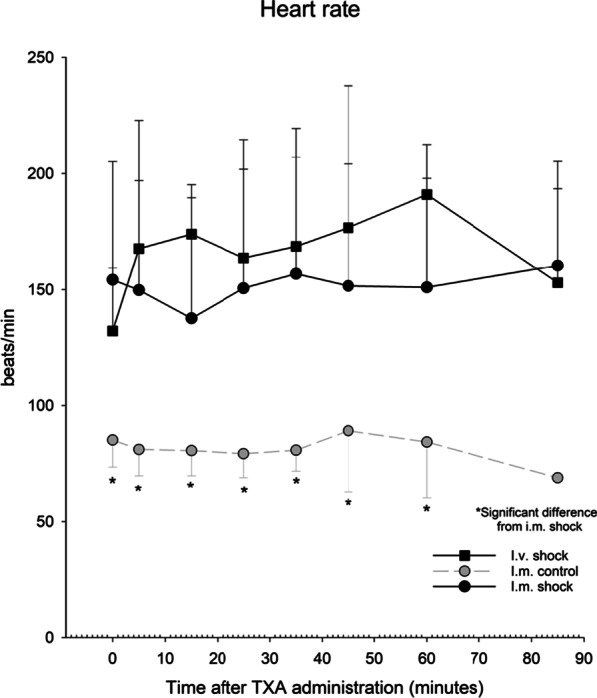
Heart rate during experiments in the three groups. *Significant difference from i.m. shock group

**Fig. 3:**
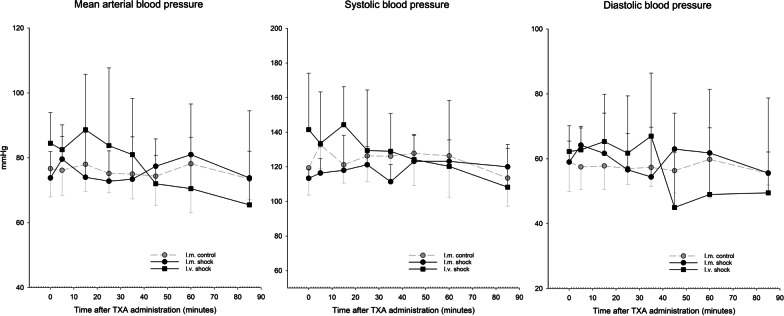
Blood pressure (mmHg) during experiments in the three groups. Mean arterial pressure (MAP) was calculated from systolic (SBP) and diastolic (DBP) blood pressure: MAP = [SBP + 2(DBP)]/3. *Significant difference from i.m. shock group

**Fig. 4:**
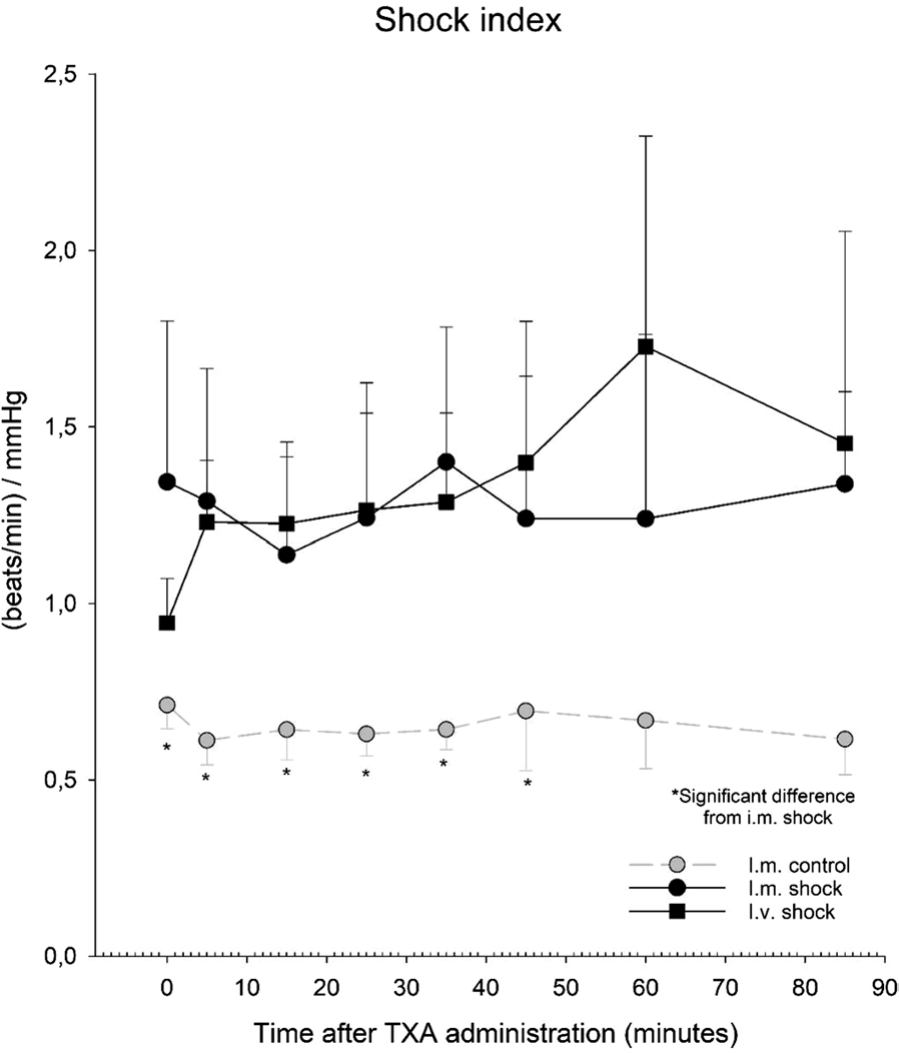
Shock index (SI) was calculated from heart rate (HR) and systolic blood pressure (SBP): SI = HR/SBP. *Significant difference from i.m. shock group

Heart rate, mean arterial pressure, diastolic blood pressure and systolic blood pressure were measured in all groups, as shown in Figs. 2 and 3. No significant changes occurred within groups during the experimental protocol. During experiments, the control group had significantly lower heart rate than the i.m. shock group, apparent already at administration, where heart rate was 85 beats per minute in the control group and 154 beats per minute in the i.m. shock group. Calculating shock index (heart rate/systolic blood pressure) showed significantly lower values in the control group, as shown in Fig. 4.

**Fig. 5:**
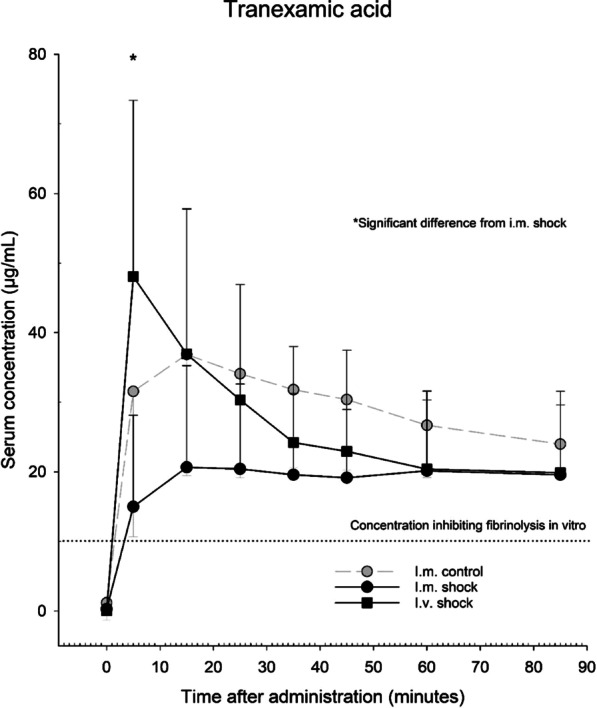
Tranexamic acid (TXA) serum concentrations were measured at pre-set time-points after administration of TXA in all experimental groups, throughout the experimental protocol. *Significant difference from i.m. shock group

In Group 1, mean maximum serum concentration was 36.9 µg/mL and occurred after 15 min. In Group 2 the mean maximum serum concentration was 48.1 µg/mL and occurred after 5 min. In group 3 mean maximum serum concentration was 20.9 µg/mL and occurred after 15 min. The temporal development of the serum concentration of TXA for the three groups are shown in Fig. 5, along with the lower end of the range in which TXA has been shown to have full effect in previous in vitro studies [3–5].

I.v. administration of TXA (group 2) resulted in C_max_ after 5 min, with significantly higher serum concentration compared to group 3. Subsequently, group 1 serum concentrations decreased significantly, already at 15 min, and remained equal to the i.m. groups throughout the remaining experimental protocol.

Both i.m. groups (1 and 3) reached C_max_ 15 min after administration. In the control group (1), TXA serum concentration was significantly reduced after 85 min, while in group 3 serum concentrations remained stable from 15 min throughout the protocol.

In all groups, mean TXA serum concentrations were above 10 µg/mL at all measured time intervals after administration.

## Discussion

The aims of this study were to determine the impact of shock on the pharmacokinetics of TXA when administered intramuscularly, and to compare the pharmacokinetics of i.v. and i.m. TXA administration in shock. We found that shock impaired muscular uptake of TXA, giving a lower C_max_, but did not affect the time to C_max_. As expected, intravenous TXA resulted in a higher C_max_. However, within 60 min following i.v. administration, serum concentrations fell to corresponding levels of TXA administered intramuscularly. TXA administered intramuscularly in shock, reached C_max_ at a serum concentration 20 ug/mL after 15 min, but thereafter kept a steady level throughout the 85 min protocol.

The serum concentrations of TXA needed to fully inhibit fibrinolysis, has been reported by in vitro studies to be in the range of 10–17.5 ug/mL, maybe even as low as 5 ug/mL [3–5]. In the present study, serum concentrations were above 10 ug/mL at all times, even after 5 min in shocked animals where TXA was administered intramuscularly. Further, in the shocked animals, serum TXA levels remained around 20 ug/mL throughout the protocol. Extrapolating to a clinical context; this suggests that TXA-treatment will achieve rapid effect if administered i.m., even in shocked patients.

Our study suggests that i.m. administration of TXA can serve as an alternative to i.v. administration. Given that i.v. administration has a quicker Cmax and retains higher concentrations the first 60 min, this should still be the standard form of administration. However, in certain situations with restraints on time or training for i.v. catheter placement, and lack of equipment for intraosseous access, i.m. administration may be an option. This might be advantageous in some military or low-resource settings [9].

Our findings are comparable to those of a recent study by Spruce et al. In a swine model, they found similar pharmacokinetics of TXA administered i.v. and i.m. under haemorrhagic shock [14]. However, their study reported mean TXA serum concentrations of 40–60 ug/mL after i.m. administration with only slightly higher doses of TXA (average 17 mg/kg against 15 mg/kg in this study). The difference may probably be attributed to the fact that Spruce et al. divided the doses of TXA in two portions, and administered one half-dose in each thigh [14]. Although it is also possible that the injuries inflicted on the animals in the present study lead to more severe shock compared to the controlled haemorrhage of Spruce et al. [14].

In an even more recent study by Grassin-Delyle et al., i.m. TXA was given to bleeding trauma patients, being the first study to do so [15]. TXA was first given as a 1 g i.v. bolus, followed by 1 g i.m., They found that therapeutic concentrations of i.m. TXA were reached within 4–11 min, which is comparable to our findings. Among their 30 included patients, they found no difference in absorption between shocked and non-shocked patients [15]. Our findings show an initial trend towards lower serum concentrations in shocked compared to non-shocked animals receiving i.m. TXA but no significant differences were detected.

Another possible implication of our finding is the prolonged, and stable effect of i.m. administration. TXA is administered as a 1 g iv. bolus followed by infusion of 1 g for a duration of 8 h [1]. In settings with limited infusion pumps, an i.v. bolus followed by an i.m. “depot”, may be an acceptable alternative to a bolus followed by i.v. infusion. This is in line with the findings of the both Spruce et al. and Grassin-Deyle, and further underpinned by Grassin-Delyle et al. reporting no adverse effects from i.m. administration among their patients [14, 15]

### Limitations

The main limitation of the study is the choice of model, where we have used swine primarily utilised for emergency trauma surgery courses. As a result, injuries, blood loss, and fluid resuscitation strategy, were not standardised. Despite this, the trends in the data were consistent. Unlike the animals in this study, most patients receiving TXA in trauma are not under general anaesthesia. General anaesthesia may influence muscular uptake of TXA [16]. The reduction of sympathetic tone may mitigate some of the negative effects shock has on muscle perfusion, and the study may underestimate the uptake compared to non-anesthetised, shocked patients. But at the same time anaesthesia itself has been shown to impair microcirculation of skeletal muscle, and may itself impair i.m. uptake [16]. Additionally, this study is limited to assess the achieved serum concentrations of TXA. And have not investigated that effect on fibrinolysis through biomarkers or viscoelastic tests. In vitro-studies reports full effect of TXA at serum levels from 5 to 17.5 ug/mL [3–5]. Viscoelastic tests from stable patients also suggests that maximum lysis inhibition is achieved somewhere below 10 ug/mL [17]. Even so, assessing whether the higher serum TXA concentrations achieved by i.v. administration translates to a superior inhibition of fibrinolysis would be of interest for future studies.

## Conclusion

We found that shock impaired muscular uptake of TXA, but even in shocked pigs, serum concentrations reached effective levels within 5 min of administration. Thus, i.m. administration of TXA may be an alternative to i.v. and intraosseous administration during stabilisation and transport to advanced medical care.

## Data Availability

All relevant data are available upon reasonable request from the corresponding author.
